# Using least angular regression to model the antibacterial potential of metronidazole complexes

**DOI:** 10.1038/s41598-021-97897-x

**Published:** 2021-09-29

**Authors:** Tahir Mehmood, Mudassir Iqbal, Bushra Rafique

**Affiliations:** 1 School of Natural Sciences, National University of Sciences and Technology (NUST), Islamabad, Pakistan; 2 Department of Chemistry, School of Natural Sciences, National University of Sciences and Technology (NUST), Islamabad, Pakistan

**Keywords:** Biochemistry, Environmental sciences, Medical research

## Abstract

Imidazole has anti-inflammatory, antituberculotic, antimicrobial, antimycotic, antiviral, and antitumor properties in the human body, to name a few. Metronidazole [1-(2-Hydroxyethyl)-2-methyl-5-nitroimidazole] is a widely used antiprotozoan and antibacterial medication. Using fourier transform infrared spectroscopy, the current study models the antibacterial activity of already synthesised Metronidazole (MTZ) complexes ($MTZ-Benz$, $MTZ-Benz-Cu$, $MTZ-Benz-Cu-Cl_2CHCOOH$, $MTZ$, $MTZ-Cu$, $MTZ-Cu-Cl_2CHCOOH$, $MTZ-Benz-Ag$, $MTZ-Benz-Ag-Cl_2CHCOOH$, $MTZ-Ag$ and $MTZ-Ag-Cl_2CHCOOH$) against *E. coli*, *B. bronceptica*, *S. epidermidis*, *B. pumilus * and *S. aureus*. To characterise the Metronidazole complexes for antibacterial activity against 05 microbes, the least angular regression and least absolute shrinkage selection operators were used. Asymmetric Least Squares was used to correct the spectrum baseline. Least angular regression outperforms cross-validated root mean square error in the fitted models. Using Least angular regression, influential wavelengths that explain the variation in antibacterial activity of Metronidazole complexes were identified and mapped against functional groups.

## Introduction

The antibacterial agent metronidazole (MTZ) [1-(2-Hydroxyethyl)-2-methyl-5-nitroimidazole] is well known. Nitroimidazoles’ biological action mechanism is based on nitro group reduction, which generates intermediate species that interact with DNA, oxidising it and liberating thymidine phosphate, resulting in a lesion characterised by double helix destabilisation and destruction^1^. The delivery of a specific and defined amount of drug to the human body via the gastrointestinal system can be accomplished in various dosage forms, such as oral tablet and suspension, among others. Various studies on the bioavailability of drugs synthesised by various manufacturers revealed that tablets and suspensions containing the same drug and drug content could not provide the same therapeutic response. The different parameters responsible for the dissolution profile and therapeutic effect variations are formulation, additives, physical form, manufacturing process, and so on. Metronidazole benzoate suspension can be used to treat infections caused by a variety of anaerobic bacteria, protozoa, and bacteroides. Metronidazole benzoate is frequently substituted for Metronidazole in paediatric oral preparations due to the blended flavour of the ester compared to the bitter taste of the free base^2^. Despite research into various ester structures, metronidazole benzoate is the only ester prodrug currently on the market. Metronidazole benzoate has demonstrated higher antimicrobial efficacy, for example, it is 20 times more effective against *Clostridium perfrigens in vitro*^3^. However, because it is almost completely insoluble in liquids, its solubility in water bodies is insufficient for injections. Solubility is a major challenge for formulation scientists. To improve the solubility of poorly soluble products, physical and chemical modifications of the material, as well as other processes such as particle size reduction, crystal engineering, salt forming, stable dispersion, surfactant application, and complexation, are all used. In this analysis, copper complexes were used to solve the solubility problem. Metal chelates had a variety of biological functions, including anticancer, antidiabetic, antibacterial, antifungal, and antiviral properties. Metal chelates have been shown to have significantly greater antimicrobial activity than chelating agents^4^. Copper acetate monohydrate can react with monomeric ligands to form an adduct by replacing the water molecules. With the split of the $[Cu_2(acetate)4(H_2O)2]$ dimer, it is even possible to lose an acetate group to allow the coordination of other ligands, or even shift the coordination of the acetate group to monodentate coordination, as with the imidazole ligand^5^. Antitumor, superoxide dismutase, and catecholase functions have been demonstrated for binuclear and mononuclear copper(II) carboxylate adducts with imidazole-type ligands^6^. These structural and electronic factors, in addition to the dependence of the structure of copper(II) carboxylates on the electronic and steric properties of added base, can influence the properties of copper(II) complexes as metalloprotein models. Nitroimidazoles are used in the prevention of bacterial infections as chemotherapeutic agents and radiosensitizers^7^.

The Cu complexes of metronidazole form a dimer in the presence of acetate due to a Cu-Cu interaction and four bridging 2-acetate ligands. Complexation improves Metronidazole’s anti-amoebic efficacy in vitro and in vivo samples, according to emphin vitro and in vivo samples^8^. The aliphatic carboxylic acid dichloroacetic acid contains only one carboxylate group (DCA). The carboxylate group in DCA can coordinate in either a monodentate or bidentate mode, according to research. Chelation can occur in bidentate or bridged systems. The researchers are interested in the synthesis, spectroscopic, computational, antimicrobial, and antidiabetic analyses of the reference drugs Metronidazole and Metronidazole benzoate with copper complexes^9^.

Fourier Transformation Infrared (FTIR) spectroscopy is used to evaluate the efficiency of Metronidazole complexes because it is simple, fast, and precise^10^. FTIR has been used in a variety of research projects. It can, for example, detect Metronidazole in environmental samples^11^ and teratogenic effects of Metronidazole on the uterus of mice^12^. FTIR assist in capturing the phenotypic screening bioassay for *Escherichia coli* stress and antibiotic responses^13^. Metronidazole’s chemical properties and antibacterial activity are characterised by FTIR using adhesive dopamine^14^.

When modelling Metronidazole antibacterial behaviour with FTIR spectral data, the sample size is much smaller than the wavelength. As a result, the data is highly dimensional (i.e. number of explanatory variables). In this case, the least absolute shrinkage and selection operator are used (Lasso )^15^ and least angle regression (LAR)^16^ are considered a viable option. The regression based algorithm are used for nondestructive pharmaceutical preparation discrimination^17^, for modeling the anitbactrial activity of ionic liquids^18,19^, in a semi-solid matrix for multivariate analysis of nystatin and Metronidazole^20^, in tablet formulations for simultaneous quantitative assessment of paracetamol and tramadol^21^, for determining the active pharmaceutical ingredient in transdermal gel formulations on a quantitative basis^22^, for predicting effective dose as a cytotoxicity biomarker^23^, for antibiotic discovery using metabolic fingerprinting and a screening assay^24^, for determining the concentrations of active ingredients in semi-solid pharmaceutical formulations^25^, for orally disintegrating tablet template formulation and non-destructive methods of assessment^26^, for anti-Helicobacter pylori drug screening^27^ and for the analysis of a binary amoxicillin-flucloxacillin mixture^28^.

The current study’s key goal is to identify functional groups for characterization of already synthesized Metronidazole complexes in terms of antibacterial activity. For this least absolute shrinkage and selection operator (Lasso )^15^ and least angle regression (LAR)^16^ are considered. FTIR spectrum was used to model the Metronidazole’s antibacterial activity, which was tested against bacterial strains *Escherichia coli (E. coli), Bordetella bronceptica (B. bronceptica), Staphylococcus epidermidis (S. epidermidis), Baccilus pumilus (B. pumilus)*, and *Staphylococcus aureus (S. aureus)*.

## Materials and methods

### Antibacterial activity of metronidazole

The data set is taken from the study^29^ where following Metronidazole complexes were synthesized ($MTZ-Benz$, $MTZ-Benz-Cu$, $MTZ-Benz-Cu-Cl_2CHCOOH$, $MTZ$, $MTZ-Cu$, $MTZ-Cu-Cl_2CHCOOH$, $MTZ-Benz-Ag$, $MTZ-Benz-Ag-Cl_2CHCOOH$, $MTZ-Ag$ and $MTZ-Ag-Cl_2CHCOOH$). The antibacterial activity of above synthesized complexes was measured against five bacteria i.e. *E. coli*, *B. bronceptica*, *S. epidermidis*, *B. pumilus * and *S. aureus*. For this Agar well diffusion process in accordance with the National Committee for Clinical Laboratory Practices’ guidelines (M07-A8) was used. The biological effects against Gram-positive and Gram-negative bacteria were examined for the screening of synthesized metal complexes for antimicrobial activity. Gram-positive strains: *S. aureus* ATCC 6538, *B. pumilus* ATCC 14884 and *S. epidermidis* ATCC 12228. Gram negative bacteria: *B. bronchiseptica* ATCC 4617, *E. coli* ATCC 8739. Microorganism cultures were grown on sterilized nutrient agar as a substrate. Surface in which microbes were grown was washed with 0.9 % saline solution to detach and obtained fresh microbial culture for further use. More over sterlization was done using autoclave and the solution was simply poured to detach from the surface microbial culture. The test sample stock solution was made in ethanol at a concentration of 1 mg/ml. Metronidazole complexes samples. The Metronidazole complexes comprising of different concentration were poured in the agar well to determine the antimicrobial activity. Initially plates were prepared containing media with microbial culture. The agar was allowed to solidify then well are punched. Afterwards, Metronidazole complexes samples dilutions were introduced in the wells. The plates were then incubated for 24 hours at 37$^oC$. The antimicrobial activities were assessed using a zone reader to measure the zone of antibacterial (mm) (Proto-COL3).

### Fourier transformation infrared spectroscopic experiment

Attenuated Total Reflection-Fourier Transformation Infrared spectroscopy was used to conduct infrared spectral measurements. The infrared spectra of the ligands and complexes were measured in solid state as KBr pellets on a PerkinElmer Spectrum 100 FT-IR Spectrophotometer in the frequency range 4000–400 $cm^{-1}$. It has a regular high linearity room temperature detector and a UATR Diamond ATR (Single Reflection). For each wavelength, 10 scans were collected, with 4 $cm^{-1}$ being the most recent resolution and 1 $cm/s$ scan speed, as used in^10^. Prior to each experiment, background spectra were obtained. The data matrix $X_{10x1676}$ is the outcome of a spectroscopic experiment with ionic liquid samples.

### Data acquisition and statistical software

The data collection studies are being carried out at the National University of Sciences and Technology (NUST) in Islamabad, Pakistan’s School of Natural Sciences (SNS). R is used for both computations and mathematical modelling. https://www.R-project.org/.

### Baseline correction

Baseline correction is part of the data preprocessing. FTIR spectra are supposed to have a straight line at 0, but this isn’t always the case due to linear or nonlinear additions. For this, asymmetric least squares (ALS) is used. For weighting down predictor variables with substantial error, it employs the least square approach and smoothing by adding a $2^{nd}$ derivative restriction^18,30^. $S=\sum w_i (x_i- b_i)^2 + \lambda \sum (\Delta ^2b_i)$

where $x_i$ is initial spectrum , $b_i$ computed baseline, $w_i$ is asymmetric residual weights, $\Delta ^2$ is the approximate baseline’s second derivative. There are two parameters to tune in the algorithm: The smoothing parameter is $\lambda$ and $w$ is the weight. Lilland’s proposed objective procedure is included in this case^31^.

### Predicting antibacterial activity

We used the least absolute shrinkage and selection operator, as well as the least angular regression, to predict the antibacterial activity of synthesised Metronidazole complexes based on FTIR spectrum data. The specifics are provided below.

#### Least absolute shrinkage and selection operator (Lasso )

Lasso^15^ is potential modeling candidate. This model employs the coordinate descent algorithm with a penalty parameter known as *theta*. Several levels are considered for computational purposes, and the optimal choice of these penalty parameters is obtained through cross validation. The partial log-likelihood function defined by is used to penalise the multivariate regression coefficient $\beta$.

1 $$\begin{aligned} L(\varvec{\beta })- \sum _{j=1}^p p_{\theta ^*}(|\beta _j|) \end{aligned}$$

where $L(\varvec{\beta })$ denotes the partial log-likelihood for $n$ samples. $p_\theta (|\cdot |)$ presents the penalty function.

In Lasso $L_1$-penalized model is used, which is defined as

2 $$\begin{aligned} p_\theta (|\beta |)=\theta | \beta | \end{aligned}$$

LASSO do the variable selection by equating non significant variable’s coefficients to zero. Here $\theta _1$ is weight for $L_1$ penalty Small $\theta _1$ level will result large number of variables and vice versa.

#### Least angular regression (LAR)

The democratic equivalent of forward step-wise Lasso regression is LAR^16^. Just as much of an indicator as LAR requires is entered. The procedure is defined as follows:

1. Start with $r=y$, $\beta _1, \beta _2, \ldots \beta _p=0$. Assume $x_l$ standardized.
2. Find explanatory variable $x_l$ most associated *r*.
3. Increase $\beta _l$ in the path of $\text{ sign }(\text{ corr }(r,x_l))$ so far some other explanatory variable $x_m$ has the same degree of correlation with the new residual as $x_l$.
4. Move $(\beta _l ,\beta _m$) in the course of the joint least squares $(x_l, x_m)$ until some other explanatory variable $x_{\ell }$ has the same degree of correlation as the new residual.
5. Continue in this manner until you’ve hit all of the predictors. Stop when $\text{ corr }(r,x_l)=0\; \forall \;l$, i.e. OLS solution.

LAR is a state of art algorithm to solve L1 regularized linear regression or logistic regression. It offers unified explanation of forward selection, stage wise regression and Lasso.

## Results and discussion

Calculations are performed for both Metronidazole as well as complexes. These complexes’ antibacterial efficacy is calculated for five bacteria: *E. coli, B. bronceptica, S. epidermidis, B. pumilus* and *S. aureus*. Table 1 shows the mean, limit, minimum, and standard deviation of antibacterial activity against the microbes studied. Synthesized Metronidazole Complexes tend to have the most activity against *S. epidermidis*, antibacterial behaviour against has shown the most difference *B. pumilus* and has lowest antibacterial activity against *B. bronceptica*.

**Figure 1:**
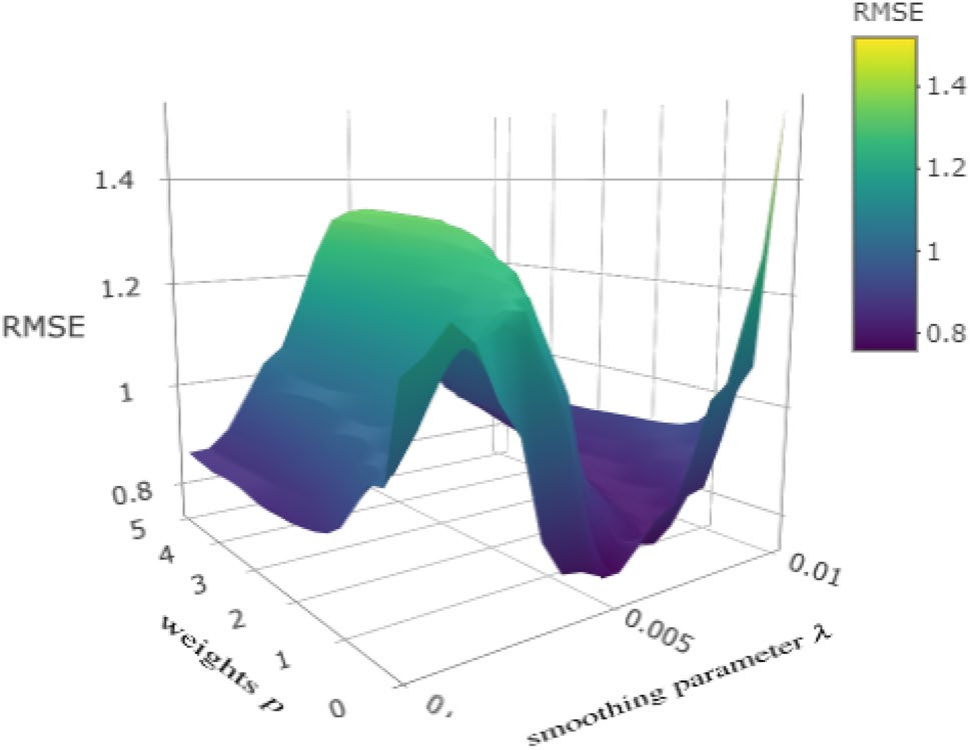
The model performance defined by RMSE of *S. aureus* with different levels of weights $p$ on x-axis and smoothing parameter $\lambda$ on y-axis is presented.

**Figure 2:**
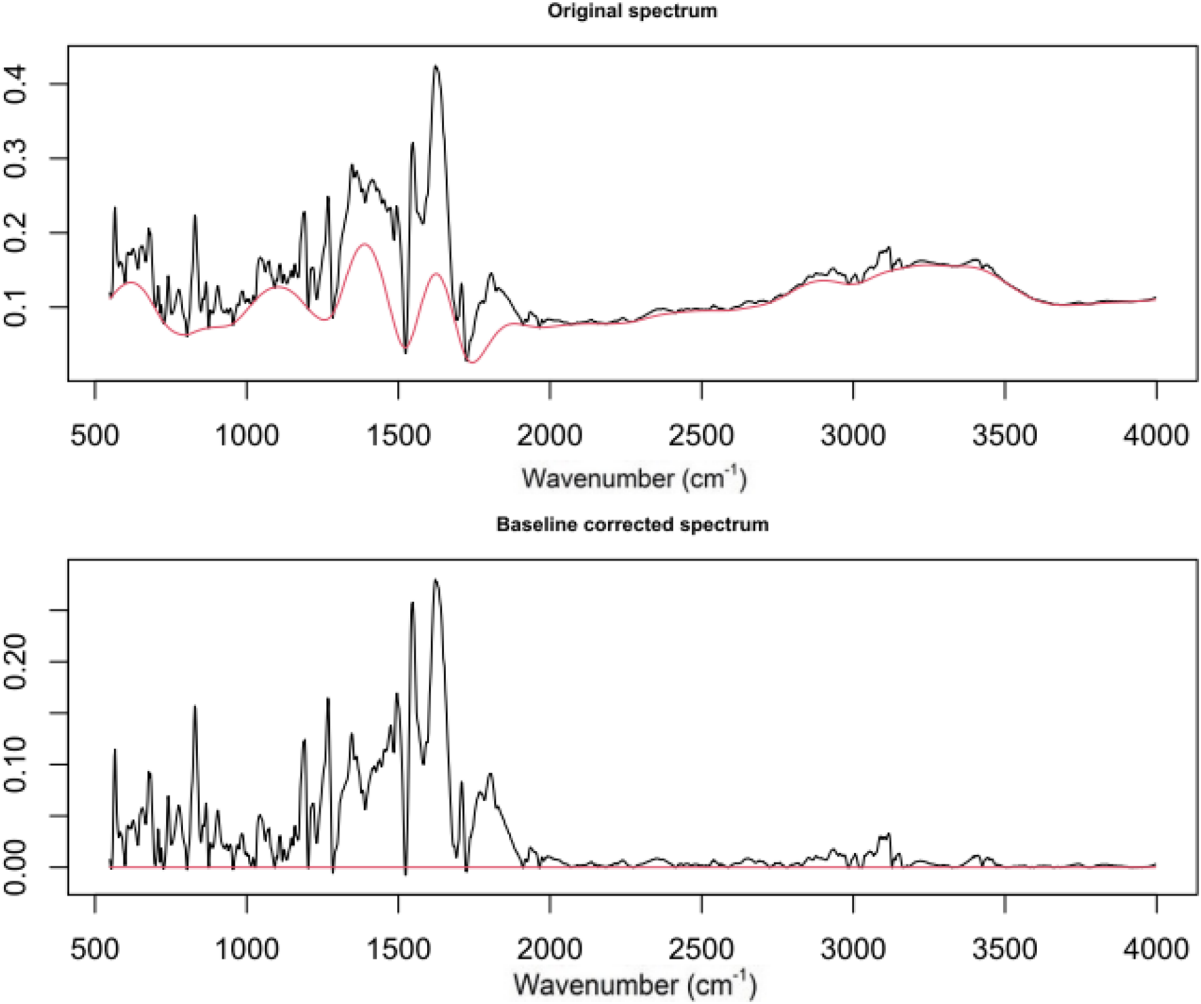
A comparison of initial and baseline corrected spectral data for complexes is shown below. With the smoothing parameter, ALS corrects the baseline for *S. aureus* $\lambda =4.100$ and wights $p=0.001$.

The ATR-FTIR spectroscopic analysis was carried out over the synthesized Metronidazole complexes samples. For identifying the influential spectrum peaks, FTIR spectrum baseline correction at zero is required. ALS is used for spectrum baseline correction. ALS has two parameters to tune, weight $p$ and smoothing parameter $\lambda$. These parameters are optimized for each fitted antibacterial activity. For in-stance, the model performance defined by RMSE of *S. aureus* with different levels of wights and smoothing parameter is presented in Fig. 1. This indicates Metronidazole antibacterial activity against *S. aureus* is optimized with $p= 0.010$ and $\lambda = 4.100$. The optimal tuned level of ALS parameters for all bacteria is presented in Table 2. By using the ALS optimal parameters for each microbe the spectrum baseline is corrected, which is further used in analysis. The compassion of original spectrum and baseline corrected spectrum obtained for *S. aureus* is presented in Fig. 2.

**Table 1:** Antibacterial activity against the bacteria under consideration is presented.

Compound	*E. coli*	*B. bronceptica*	*S. epidermidis*	*B. pumilus*	*S. aureus*
$MTZ-Benz$	22.09	19.37	25.09	25.93	18.29
$MTZ-Benz-Cu$	20.25	19.01	22.90	21.47	13.22
$MTZ-Benz-Cu-Cl_2CHCOOH$	21.81	22.77	23.72	20.87	16.01
$MTZ$	15.72	17.72	14.32	13.64	10.09
$MTZ-Cu$	16.09	19.09	15.51	14.38	14.72
$MTZ-Cu-Cl_2CHCOOH$	16.90	19.90	16.36	16.98	14.77
$MTZ-Benz-Ag$	20	19	21	24	17
$MTZ-Benz-Ag-Cl_2CHCOOH$	19	20	22	22	16
$MTZ-Ag$	18	18	23	20	15
$MTZ-Ag-Cl_2CHCOOH$	17	17	21	23	14

**Table 2:** For LASSO and LAR, the distribution of ALS parameters that define the baseline adjustment, shrinkage parameters that define the wave-number selection, and RMSE is shown.

Response	ALS parameters		Shrinkage parameter		CVRMSE	
	P	$\lambda$	$\theta$	$fraction$	LASSO	LARS
*E. coli*	0.001	2.679	1.8	0.02	2.38	2.32
*B. bronceptica*	0.001	0.616	1.3	0.06	1.97	1.91
*S. epidermidis*	0.010	0.100	1.1	0.07	2.33	2.01
*B. pumilus*	0.001	4.226	1.6	0.01	1.84	1.84
*S. aureus*	0.010	4.100	1.5	0.02	2.14	1.91

**Table 3:** By using the bootstrapping with LAR consistent influential wavenumbers for *B. bronceptica, E. coli, S. epidermidis, B. pumilus and S. aureus* are selected. The best-fitted model’s powerful regression coefficients for each bacteria are presented. Furthermore, for each model, the influential compound assignment and respective group is indicated.

Response	Influential wavenumbers ($cm^{-1}$)	Functional compounds	Functional group
*E. coli*	1802	$C=O$	Carboxylic acids and derivatives
	2930	$CH_3$, $CH_2$, *CH*	Alkanes
	3156	$C-H$	Carboxylic acids and derivatives
*B. bronceptica*	1475	$CH_2$, $CH_3$	Alkanes
	135	$C=O$	Carboxylic Acids and Derivatives
	2999	$CH_3$, $CH_2$, *CH*	Alkanes
*S. epidermidis*	2246	$C\equiv C$	Alkynes
	2670	$O-H$	Carboxylic acids and derivatives
*B. pumilus*	1298	$O-C$	Carboxylic acids and derivatives
	2545	$O-H$	Carboxylic acids and derivatives
	3010	$CH_3$, $CH_2$, *CH*	Alkanes
*S. aureus*	930	$=C-H$, $=CH_2$	Alkanes
	2845	$O-H$	Carboxylic acids and derivatives
	3636	$O-H$	Alcohols and phenols

**Figure 3:**
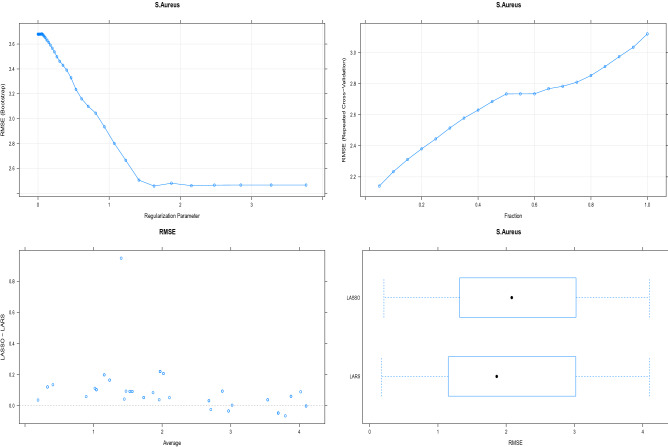
RMSE is used to assess the ability of a model to predict MTZ antibacterial activity against *S. aureus*. In the upper left and right panels, the RMSE behaviour of LASSO and LAR against their regularized parameter (theta) and fraction. We used bootstrap cross validation for model fitting, comparing the model RMSE in each fold.

LASSO and LAR are used to model Metronidazole’s antibacterial activity against the microbes in question. Regularized parameters $\theta$ and fraction respectively determine the efficiency of LASSO and LAR . The change of these regularization parameters define the model prediction capability but also effects the number of wave-number selection. These parameters are optimized for prediction capability measured by RMSE in upper panel of Fig. 3 for *S. aureus*. For lasso, the optimal shrinkage parameter $\theta$ is tuned to 1.5 and for LAR the optimal shirnkage parameter $fraction$ is tuned to 0.02 which results the RMSE 2.14 and 1.91 respectively. For model fitting we have used bootstrap cross validation, at each fold the model RMSE was computed and is compared in lower panel of Fig. 3. The lower left panel indicates LAR has lower RMSE in most of the folds when compared to LASSO. Similar findings are found in lower right panel indicating LAR has better prediction capability of modeling antibacterial activity of Metronidazole against *S. aureus*. The distribution of ALS parameters which defines the baseline correction, the shrinkage parameters which defines the wave number selection and RMSE for LASSO and LAR is presented in Table 2. For prediction of antibacterial activity of Metronidazole for *E. coli*, LASSO uses $\theta =1.8$ to optimizes the RMSE= 2.38 and LAR uses $fraction=0.02$ to optimizes RMSE= 2.32. For prediction of antibacterial activity of Metronidazole for *B. bronceptica*, LASSO uses $\theta =1.3$ to optimizes the RMSE= 1.97 and LAR uses $fraction=0.06$ to optimizes RMSE= 1.91. For prediction of antibacterial activity of Metronidazole for *S. epidermidis*, LASSO uses $\theta =1.1$ to optimizes the RMSE= 2.33 and LAR uses $fraction=0.07$ to optimizes RMSE= 2.01. For prediction of antibacterial activity of Metronidazole for *B. pumilus*, LASSO uses $\theta =1.6$ to optimizes the RMSE= 1.84 and LAR uses $fraction=0.01$ to optimizes RMSE= 1.84. LAR is predicting the antibacterial activity of Metronidazole complexes as good as LASSO or in some cases it is better. Hence we have used LAR for extracting the influential wavenumbers. LAR computes the importance index for each variable that is wavenumebr. Since LAR is defined by the shrinkage parameter $fraction$, see Table 2. We have extracted the wavenumebrs by using the optimally tuned $fraction$ in LAR for each microbial model. We have used repeated sampling based modeling that is bootstrap where the consistently selected LAR coefficients are used to identify the influential wave numbers. The influential wavenumbers are mapped for respective functional compound and group, and are presented for each microbes in Table 3.

For *E. coli* the influential wavenumbers are mapped to the functional compound $C-H$, $CH_3, CH_2, CH$, $C=O$ and $O-H$, which respectively belong to Carboxylic Acids & Derivatives, Alkanes, and Carboxylic Acids & Derivatives functional group. For *B. bronceptica* the influential wavenumbers are mapped to the functional compound $CH_2, CH_3$, $C=O$ and $CH_3, CH_2, CH$ which respectively belong to Alkanes, Carboxylic Acids & Derivatives and Alkanes. For *S. epidermidis* the influential wavenumbers are mapped to the functional compound $C \equiv C$ and $O-H$, which respectively belong to Alkenes and Carboxylic Acids & Derivatives functional group. For *B. pumilus * the influential wavenumbers are mapped to the functional compound $O-H$, $O-C$ and $CH_3, CH_2, CH$, which respectively belong to Carboxylic Acids & Derivatives and Alkenes functional group. For *S. aureus* the influential wavenumbers are mapped to the functional compound $O-H$, $=C-H, =CH_2$ and $O-H$, which respectively belong to Alcohols & Phenols, Alkenes, and Carboxylic Acids & Derivatives functional group.

### Computations

For computations, modeling and figures R software is used^32^. For baseline correction R package ’baseline’^33^ and for LASSO and LAR model fitting R package ’caret’^34^ is used.

## Conclusions

The aim of this study is to find functional groups for characterization of already synthesised Metronidazole complexes with regard to antibacterial activity. The least absolute shrinkage and selection operator (Lasso ) and least angle regression (LAR) was used to model the antibacterial behaviour of Metronidazole complexes using the FTIR spectrum, where the antibacterial activity was tested against bacterial strains *E. coli, B. bronceptica, S. epidermidis, B. pumilus and S. aureus*. The practical compound’s powerful group is also mapped. In addition, research is needed to analyses and examine existing findings for medical applications. Moreover in future work, we aimed to identify the cluster of influential wavenumbers which can better characterize the Metronidazole complexes.

## Supplementary Information

Supplementary Information.
